# Self‐reported rates of impulsivity in Parkinson’s Disease

**DOI:** 10.1002/acn3.51016

**Published:** 2020-03-29

**Authors:** Megan A. Aumann, Adam J. Stark, Shelby B. Hughes, Ya‐Chen Lin, Hakmook Kang, Elise Bradley, David H. Zald, Daniel O. Claassen

**Affiliations:** ^1^ Vanderbilt Brain Institute Department of Psychology Vanderbilt University Nashville Tennessee; ^2^ Department of Neurology Vanderbilt University Medical Center Nashville Tennessee; ^3^ Department of Biostatistics Vanderbilt University Medical Center Nashville Tennessee; ^4^ Department of Psychiatry Vanderbilt University Medical School Nashville Tennessee; ^5^ Department of Psychology Vanderbilt University Nashville Tennessee

## Abstract

**Objective:**

Impulsive decision‐making is characterized by actions taken without considering consequences. Patients with Parkinson's disease (PD) who receive dopaminergic treatment, especially dopamine agonists, are at risk of developing impulsive–compulsive behaviors (ICBs). We assessed impulse‐related changes across a large heterogeneous PD population using the Barratt impulsivity scale (BIS‐11) by evaluating BIS‐11 first‐ and second‐order factors.

**Methods:**

We assessed a total of 204 subjects: 93 healthy controls (HCs), and 68 ICB– and 43 ICB + PD patients who completed the BIS‐11. Using a general linear model and a least absolute shrinkage and selection operation regression, we compared BIS‐11 scores between the HC, ICB– PD, and ICB + PD groups.

**Results:**

Patients with PD rated themselves as more impulsive than HCs in the BIS‐11 total score, second‐order attention domain, and first‐order attention and self‐control domains. ICB + patients recorded higher total scores as well as higher scores in the second‐order non‐planning domain and in self‐control and cognitive complexity than ICB– patients.

**Interpretation:**

These results indicate that the patients with PD show particular problems with attentional control, whereas ICB + patients show a distinct problem in cognitive control and complexity. Additionally, it appears that all patients with PD are more impulsive than their age‐ and sex‐matched healthy peers. Increased impulsivity may be a result of the disease course, or attributed to dopaminergic medication use, but these results emphasize the importance of the cognitive components of impulsivity in patients with PD.

## Introduction

Impulsivity is a multifaceted construct, involving several factors including quick action, lack of focus on tasks, and lack of planning,^1^ and is expressed behaviorally, via actions in daily life, and/or through performance on cognitive assessments.^2^, ^3^, ^4^ Impulsivity is generally thought to include a lack of behavioral inhibition and/or premature decision‐making, and when it becomes a behavioral problem, for example, impulse control disorders, can manifest through engagement of spontaneous, unplanned, or reckless activities regardless of potential negative consequences.^5^, ^6^ Maladaptive impulsivity is a feature of several neuropsychopathologies, including attention‐deficit/hyperactivity disorder, borderline personality disorder, and substance abuse.^7^

Poor proficiency of impulse control is common in patients with Parkinson’s disease (PD), in which dopamine therapy is the standard of care in treating the motor movement disruptions resulting from progressive degeneration of dopamine neurons in the substantia nigra pars compacta. However, PD is a complex disease, impacting cognitive, behavioral, and emotional symptoms, all of which need to be considered when determining personalized treatment plans.^8^, ^9^, ^10^ Impulsive–compulsive behaviors (ICBs) have gained recent attention in the literature with estimates of above 25%^10^, ^11^ in patients with PD being treated with dopamine agonists (DAAs). Patients with PD who take DAAs, such as pramipexole and ropinirole, show marked improvements in their motor symptom severity.^12^ However, a subset of patients with PD taking these agonists have been reported to develop maladaptive ICBs such as pathological gambling, shopping, binge eating, and hypersexuality, as well as heightened novelty seeking.^13^, ^14^, ^15^, ^16^ Impulsive shifts that occur in PD may be underappreciated by patients who are experiencing a multitude of changes in their lives as part of their disease. Assessing their subjective experiences of behavioral and cognitive control can give caregivers and treatment providers insight into some of the earlier changes that may precede development and expression of an ICB.

Although the Questionnaire for Impulsive‐Compulsive Disorders in Parkinson’s Disease (QUIP) is used often as an instrument for assessing impulsive behaviors in PD, it lacks broader cognitive constructs such as attention and planning, which are known to be altered in patients with PD.^4^, ^17^, ^18^, ^19^, ^20^ Furthermore, the scope of ICBs in PD encompasses a broader range of compulsive appetitive behaviors such as hypersexuality, compulsive shopping, gambling, and medication use.^5^, ^21^ Although these are troublesome problems, the QUIP does not capture impulsive behavioral changes that may occur outside of the conventionally defined features of ICB.

**Figure 1:**
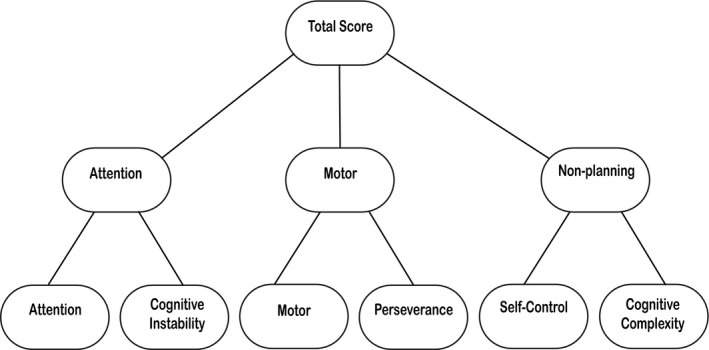
BIS‐11 hierarchy structure. Each of the six first‐order factors in the bottom row contribute to two factors of the broader second‐order factors (attention, motor, and non‐planning) in the middle row. Each of the three second‐order factors contributes to the total score.

The Barratt impulsivity scale (BIS‐11)^1^ is a common self‐report instrument used to assess impulsivity, and has been used in a variety of populations.^22^, ^23^, ^24^, ^25^, ^26^ It is designed to assess the behavioral construct of impulsivity through 30 items that describe cognitive and behavioral preferences. The BIS‐11 provides information not only about overall impulsivity through a total score but also on the more specific facets of impulsivity through the first‐ and second‐factor subscales. There are six first‐order factors (attention, cognitive instability, motor, perseverance, self‐control, and cognitive complexity) and three second‐order factors (attention, motor, and non‐planning), with two first‐order factors forming each second‐order factor. For instance, the first‐order factors, attention and cognitive instability, form the second‐order attention domain, motor and perseverance form the motor domain, and self‐control and cognitive complexity form non‐planning (Fig. 1). Although many studies of PD report BIS‐11 total scores, and some of which report second‐order scores,^6^, ^15^, ^27^, ^28^, ^29^ none to our knowledge have investigated first‐order factors. Although these studies do tend to find differences in total and second‐order level BIS‐11 scores, there are inconsistencies on how second‐order factors differ between PD and healthy participants. These may be due to poor statistical power, given frequent sample sizes less than 100. A larger cohort of patients with PD would allow for an in‐depth look at both the first‐ and second‐order subscales, elucidating both the primary traits expressed in patients with PD, as well as those who meet criteria for ICB. Assessing first‐order factor data could impart a more detailed understanding of impulsive changes within a PD population.^27^ For instance, both the first‐order factors, attention and cognitive instability, contribute to the second‐order factor, attention, but they are comprised of different elements; the first‐order factor (attention) reflects a failure to maintain cognitive attention, while the first‐order factor (cognitive instability) is characterized by the presence of racing or extraneous thoughts. By analyzing distinct factors of impulsiveness, we hope to understand the nature of self‐reported ratings of impulsivity in PD, and especially in patients with ICBs.

In this study, we applied the BIS‐11 to a large number of participants with PD and without PD. We assessed the relative contribution of the total, first‐order, and second‐order factors, as well as the contribution of individual questions from the BIS‐11. We also assessed the precise relationships of self‐reported impulsivity in ICB + and ICB– PD patients.

## Materials and Methods

### Subjects

A total of 204 participants completed the BIS‐11 and a clinical interview (Table 1). All healthy participants were recruited from the Nashville, TN area and patients with PD were recruited from the Vanderbilt University Movement Disorders Clinic. PD recruitment efforts were not biased toward a single subcategory of behaviors. All participants provided a written informed consent approved by the Vanderbilt University Institutional Review Board. The diagnosis of PD was based on the United Kingdom Brain Bank criteria,^30^, ^31^ and patients with PD meeting this criterion were prescribed levodopa/carbidopa and/or DAA for relief of motor symptoms. Patients were excluded if they had implanted deep brain stimulator, received antipsychotic treatments, suffered from comorbid neuropsychiatric, cerebrovascular, or cardiovascular disease (as determined through medical history, and clinical interview). Healthy control (HC) subjects did not have a history of psychiatric illness, head trauma, substance abuse, or comorbid vascular disease. The Unified Parkinson’s Disease Rating Scale (UPDRS) examination was performed on all participants to rate symptom severity in population with PD, and a neurologic assessment was used to confirm the absence of parkinsonian features in HC subjects.^32^

**Table 1:** Demographic information based on the population groups (HC, PD ICB–, and PD ICB+).

Variable	HC	PD ICB–	PD ICB+	Statistic*	*P*‐value	Tukey post hoc
*N*	93	68	43	–	–	
Gender (male/female)	50/43	53/15	25/18	10.31^1^	0.01	
Age (years)	57.96 (7.98)	64.97 (8.22)	60.98 (6.97)	15.22^2^	<0.01	0.001^A^
Disease duration (years)	–	5.01 (3.72)	4.07 (2.62)	2.02^2^	0.16	
MoCA score	–	25.38 (2.69)	26.67 (2.36)	6.31^3^	0.01	
UPDRS						
II	–	20.59 (9.27)	21.06 (8.11)	0.06^3^	0.81	
III	–	27.58 (12.35)	25.88 (13.06)	0.39^3^	0.54	
Dopamine replacement therapy						
Total LEDD (mg/day)	–	740.95 (410.6)	642.59 (397)	1.27^3^	0.26	

Gender is shown as the ratio of males to females. Scores for age, disease duration, MoCA, UPDRS II, UPDRS IIII, and total LEDD are shown as averages with standard deviations in parentheses.

^1^ indicates the chi‐squared test.

^2^ indicates the *F*‐value for t‐tests.

^3^ indicates the *F*‐statistic for an ANOVA. The superscript A indicates a significant difference between the HC and ICB– groups.

* Different statistical tests were performed for the data where the superscript number indicates the test used.

The presence of ICB was determined by a clinician and defined as clinically problematic behavior(s) following DAA treatment according to the Diagnostic and Statistical Manual of Mental Disorders (DSM‐V).^33^

### Experimental task and procedures

Patients with PD completed part II of the UPDRS (questionnaire of patient‐rated motor experiences of daily living) and part III (a clinical assessment of motor function in PD) in an OFF‐medication condition after overnight washout of dopamine medications.^34^, ^35^ Healthy controls were deemed free of motor deficits through medical history and neurologic examination by a physician.

All participants completed the self‐report BIS‐11 questionnaire^36^, ^37^ (ON‐medication for PD subjects), which uses a 4‐point Likert‐type scale: rarely/never, occasionally, often, and almost always/always. We determined the total score as well as separate scores for the six first‐order factors and the three second‐order factors. The Montreal Cognitive Assessment (MoCA) was administered to assess PD patients’ global cognitive abilities, and to exclude individuals who were severely impaired.^38^, ^39^ MoCA scores range from 0 to 30, with higher scores indicating better cognitive function. Considering the age range of the sample for this study, we excluded patients with a score of 22 or below on the MoCA examination.^40^, ^41^, ^42^, ^43^ Healthy controls were initially recruited for the purpose of a separate study and therefore did not complete a MoCA but were deemed cognitively intact without any evidence of cognitive impairment or neuropsychiatric disorder through a battery of neuropsychological assessments (e.g.,Stroop task^44^, Wechsler Adult Intelligence Scale,^45^ and Structured Clinical Interview for the DSM^46^).

### Data analysis

Differences in group demographics were determined by a *t*‐test or ANOVA for comparing all three groups. Sex differences between the groups were tested using the chi‐square test. For demographic information, values of *P* < 0.05 were considered significant (Table 1). A general linear model (GLM) controlling for age and gender followed by a false discovery rate (FDR) correction was used to analyze the group mean differences for HCs and PD participants with and without ICB (ICB+/ICB–) with a threshold for significance set at *P* < 0.05 using R statistical software version 3.5.2 (R Foundation for Statistical Computing, Vienna, Austria). For each GLM, the *t*‐statistic for the variable of interest only was reported and *P* value was computed accordingly. The FDR correction was performed on the computed *P*‐values as described in the original FDR paper;^47^ FDR corrections were performed on multiple applications of GLM and not after each GLM. All BIS‐11 *P*‐values shown were corrected for FDR at 0.1.

The BIS‐11 presents interpretational challenges due to concerns about the fit of factor solutions, redundancy of some questions, and low correlations between others.^48^ To address impulsivity in our cohort without the constraints of *a priori* first‐ and second‐order scales, we applied a least absolute shrinkage and selection operation (LASSO) regression to observe group responses to individual questions of the BIS‐11 with 500 bootstraps, controlling for age, gender, and disease duration in participants with PD.^49^ This approach simultaneously performs regularization and variable selection, which allows for a higher prediction accuracy and specificity of interpretation. The variable with ≥ 80% chosen is deemed significant in relation to either PD/HC or ICB+/– status.^50^, ^51^ LASSO regression was performed using the glmnet package and bootstrapped in R statistical software.^52^

## Results

### Demographics

Both the PD ICB– and PD ICB + groups had significantly more males than females (*t* = 10.31, *P* = 0.01). The HC group had significantly younger population than the PD ICB– and PD ICB + groups (*t* = 15.22, *P* < 0.01). Among our participants with PD, there was no significant difference in the overall disease duration between the ICB– and ICB + groups (*t* = 2.02, *P* = 0.128). There were no significant differences in average UPDRS II or III scores between ICB– and ICB + groups (*t* = 0.06, *P* = 0.39 and *t* = 0.39, *P* = 0.27, respectively). There was a significant decrease in MoCA scores in the ICB– patient group compared to ICB + patients (*t* = 6.31, *P* = 0.01).

### BIS‐11

#### Total score

**Figure 2:**
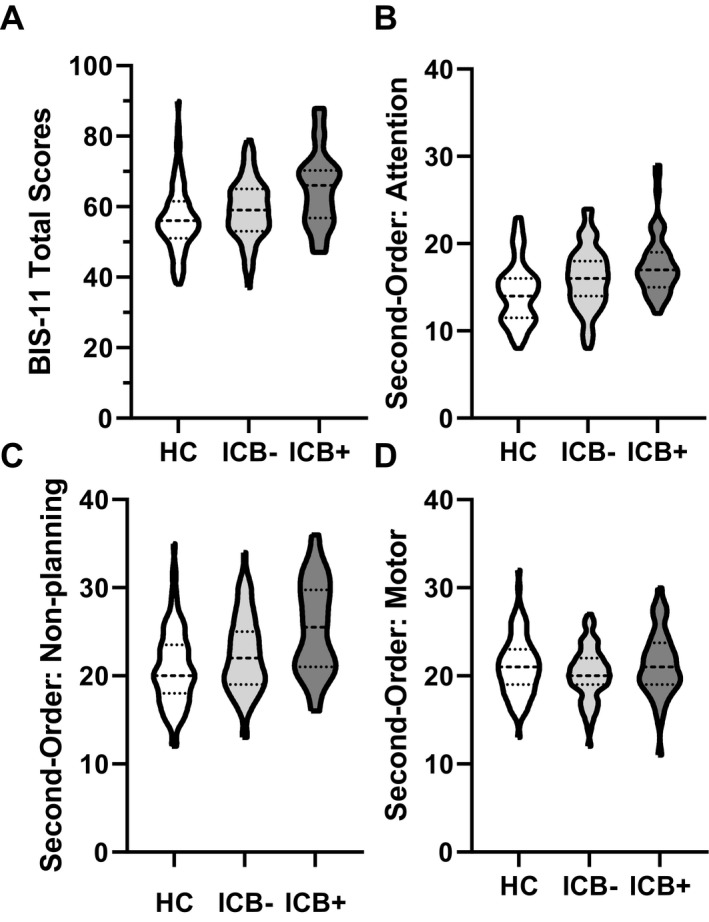
Violin plots show the group responses for the BIS‐11 total score (A), and second‐order factors: attention, motor, and non‐planning (B–D). The thickest dashed line in the middle of each violin plot indicates the median.

The BIS‐11 total scores increased in a stepwise fashion (Fig. 2), where the HC group scored the lowest, PD ICB– group scored significantly higher than the HC group (*t* = 2.49, $P_{\text{corr}}$ = 0.045), and the PD ICB + group scored significantly higher than the PD ICB– (*t* = 2.40, $P_{\text{corr}}$ = 0.045) and HC groups (*t* = 5.63, $P_{\text{corr}}$ <0.001) (Fig. 2A).

#### Second‐order factors

Both the PD ICB– and PD ICB + groups scored significantly higher than the HC group in the attention domain (*t* = 2.52, $P_{\text{corr}}$ = 0.045, and *t* = 3.33, $P_{\text{corr}}$ = 0.008, respectively; Fig. 2B). Additionally, both the PD ICB– and PD ICB+ groups scored significantly higher on average than the HC group in the non‐planning domain (*t* = 2.75, *P* = 0.007; *t* = 3.65, $P_{\text{corr}}$ = 0.0003, respectively; Fig. 2C). There were no significant differences in the motor domain (Fig. 2D).

#### First‐order factors

**Figure 3:**
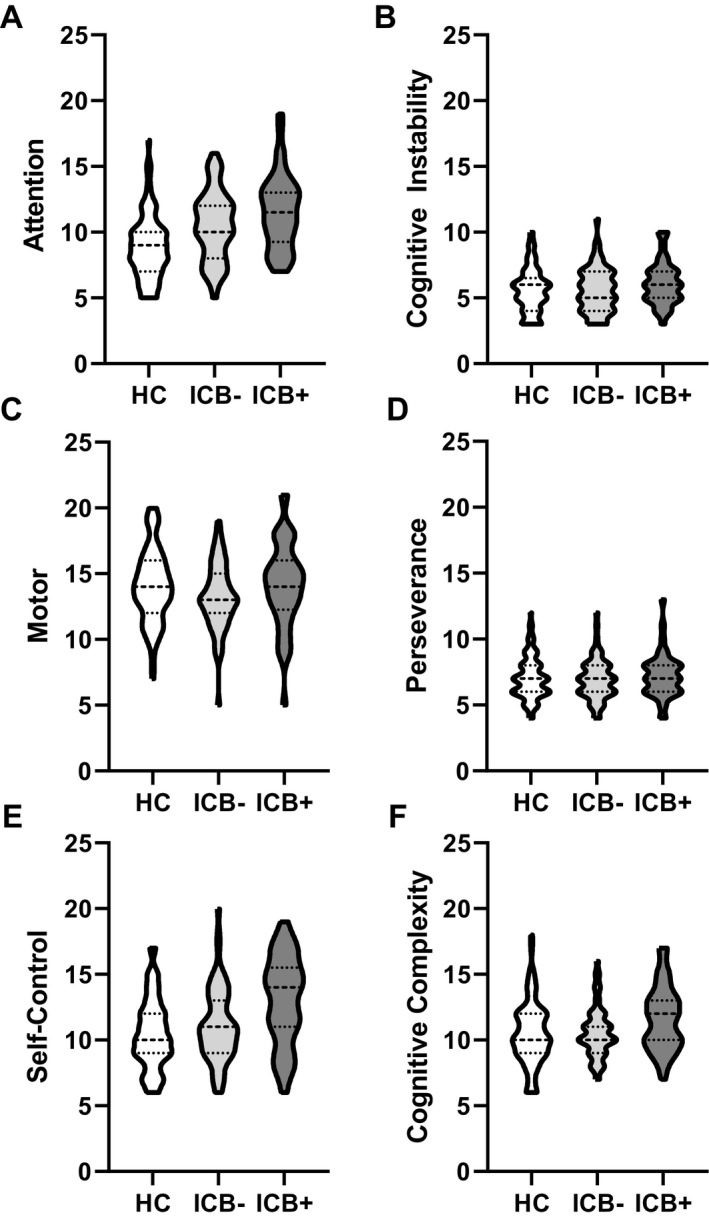
Violin plots show the group results for each of the first‐order factors: attention (A), cognitive instability (B), motor (C), perseverance (D), self‐control (E), and cognitive complexity (F). The thickest dashed line in the middle of each plot indicates the median.

The PD ICB + group scored significantly higher than the HC group in the attention (*t* = 4.07, $P_{\text{corr}}$ < 0.001), self‐control (*t* = 3.78, $P_{\text{corr}}$ < 0.001), and cognitive complexity (*t* = 3.42,  = 0.003) domains (Fig. 3A, 3E, and F, respectively). The PD ICB– group also scored significantly higher than the HC group in the attention (t = 2.52, < 0.001) and self‐control (t = 2.35, $P_{\text{corr}}$ = 0.048) domains, such that a stepwise pattern emerges in which both average attention and self‐control scores increase from HC to PD ICB– and to PD ICB+ (Fig. 3A and E). The cognitive instability, motor, and perseverance domains showed no significant differences between any groups. When we run the GLM model controlling for MoCA scores, the first‐order factor attention is no longer significant between ICB– and ICB + subjects (for further detail, see Table S2).

#### LASSO Regression

**Figure 4:**
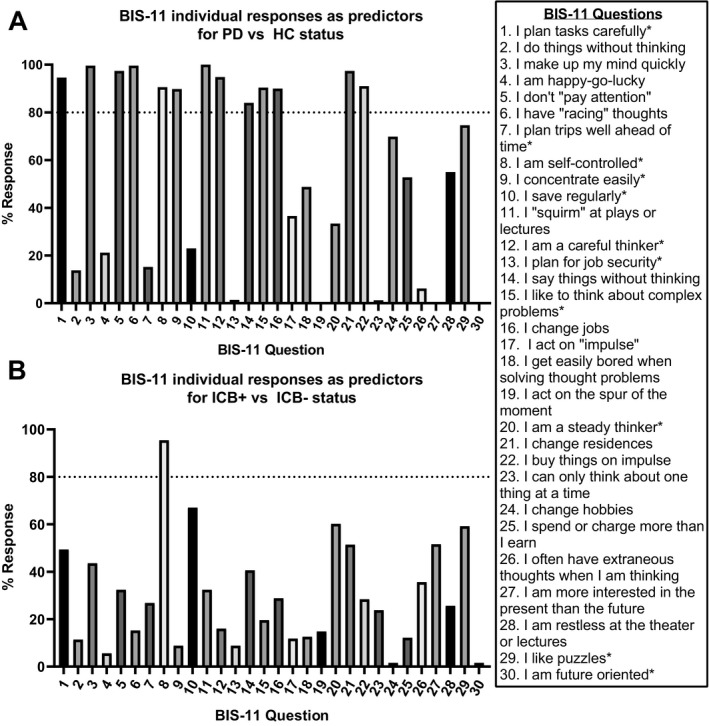
LASSO graphs show the frequency where BIS‐11 question is marked as "always/almost always" (unless reversed scored as indicated by a * symbol) for PD versus HC (A) or for PD ICB+ versus PD ICB– (B).

A LASSO regression shows how responses to individual questions contribute to outcomes, in this case, disease status (HC, IBC–, and ICB+). The LASSO analysis identified 13 individual questions from the BIS‐11 that were chosen with a frequency of ≥ 80% as important questions for distinguishing between the HC and PD state. Subjects with PD were more likely to respond with “Almost Always,” unless the question is starred, in which case subjects with PD were more likely to report “Rarely/Never” (Fig. 4A). Additionally, when looking at questions that distinguish between ICB status (ICB+/–), for example, question 8, “I am self‐controlled,” is more likely to distinguish ICB+ subjects, who more reported “Almost Always” with a frequency of ≥ 80% (Fig. 4B). It may be worth noting that if you extend the threshold to a choice of ≥ 60%, two more questions emerge as important distinguishers between ICB status (“I save regularly” and “I am a steady thinker”).

## Discussion

Cognitive and behavioral changes that impact motivation and attention are common features in patients with PD, especially as the disease progresses.^51^, ^53^, ^54^, ^55^, ^56^, ^57^ In a large PD cohort, we have demonstrated that patients report behavioral symptoms linked to elevated impulsivity. This increase is independent of a diagnosis of ICB, with symptoms primarily in the attention and non‐planning domains and occurring at elevated rates in both the PD patient groups. Patients with clinically diagnosed ICBs report even greater BIS‐11 scores in these domains. A question‐based regression analysis highlights that ICB patients experience a perceived lack of self‐control. Importantly, the study results emphasize that patients with PD are subjectively aware of changes to self‐regulation of behavior and thinking, and that the nature of these deficits are heightened in patients with ICB.

These results are consistent with previous studies that define changes to delayed discounting, and altered reward strategies, in patients with ICBs.^6^, ^15^, ^27^, ^29^, ^58^ Interestingly, we observed significant differences between the ICB+ and ICB‐ groups in only one first‐order factor (attention) and in the second order factor non‐planning (self‐control and cognitive complexity). The attention domain reflects an inability to focus or concentrate on a specific task, while the non‐planning domain reflects an inability to defer gratification, where patients note difficulty in either staying focused enough to complete a task, or struggle with strategic decisions that require delayed gratification. These results are consistent with previous studies assessing delayed discounting and reward strategies,^23^, ^59^, ^60^ although this effect was not apparent in a smaller cohort,^61^ nor a cohort that did not compare scores to a group of HCs.^62^ Furthermore, while previous studies focus on PD patients with and without ICB, we included analyses that self‐assess behavioral symptoms in a healthy cohort. Findings regarding increased motor impulsivity are less consistent, with some studies showing increased motor impulsivity in ICB patients,^15^, ^61^ There are no elevations in BIS‐11 motor impulsivity scores and this is consistent with previous studies that show intact behavioral motor inhibitory control in ICB patients ^63^, ^64^, ^65^ and lower self‐reported ratings of motor impulsivity in PD patients with addictions.^27^

Our results emphasize that impulsive behavioral changes occur in PD, regardless of ICB status. Indeed, the LASSO analysis reveals most questions distinguishing PD from HCs that align with the changes to attention or inhibitory control, as demonstrated by high ratings on questions such as: “I don’t ‘pay attention’,” “I (don’t) plan tasks carefully,” “I am (not) self‐controlled,” and “I buy things on impulse.” Although the Urgency‐Premeditation‐Perseverance‐Sensation seeking Impulsive Behavior Scale (UPPS) measures different dimensions of impulsivity than the BIS‐11, our findings align well with the overall findings from a study that found that subjects with PD had lower premeditation and greater risk taking than HCs.^66^ While it is difficult to compare BIS‐11 findings, we believe that the changes in self‐control and cognitive complexity agree with this finding. Of note, a few questions did not appear to align well with the cohort demographics, such as “I change jobs” and “I change residences.” Conceivably, these questions may not be relevant to an older cohort and could be modified or excluded in future studies in an older population.

Previous attempts to describe the pattern of cognitive changes that evolve over the course of PD suggest a progression from anterior (attention and executive function) to posterior (visuospatial and memory) dysfunction.^56^, ^67^, ^68^, ^69^, ^70^ Cognitive deficits are present at various stages of disease, including in the prodromal stage, and early in the disease course.^71^ Impairments to attention and planning are likely a result of alterations to frontostriatal circuitry, where the anterior cingulate cortex (ACC; i.e., response initiation, intention, and inhibition),^51^, ^72^, ^73^, ^74^ orbitofrontal cortex (OFC; decision‐making and encoding values of expected reward outcomes),^51^, ^74^, ^75^ and dorsolateral prefrontal cortex (DLPFC; complex problem‐solving, organizational planning strategies, concept‐formation, and working memory)^76^ that are functionally linked to basal ganglia structures are altered in PD. Our findings agree with previously described changes to frontostriatal networks, where behavioral impulsive actions, reflected in items such as “I (don’t) plan tasks carefully,” “I say things without thinking,” and “I buy things on impulse,” reflect challenges with exerting behavioral self‐control. Interestingly, when we rerun our model additionally controlling for MoCA scores between the ICB– and ICB + groups, both the first‐order factors (self‐control and cognitive complexity) remain significant, but the first‐order factor (attention) is no longer significant between these groups. These findings suggest that increased impulsivity may be a direct consequence of deteriorating cognitive function. It may be noted that patients with MoCA score of less than 22 were excluded. Although patients with dementia were excluded, in the absence of formal neuropsychological testing, it is possible that some patients may have met criteria for mild cognitive impairment. A previous study found that there was no difference in BIS‐11 scores and domain scores between PD and PD‐MCI patients.^66^ We hypothesize that self‐reported problems with attention in this population with PD may reflect early dysexecutive symptoms, of which the MoCA screening is heavily weighted. Behavioral changes linked to attentional and executive dysfunction should be formally explored in future studies assessing cognitive decline and behavioral impulsivity in PD. Our findings emphasize that impairments to self‐regulation are a key deficiency in the ICB population. While self‐report measures of impulsivity often have only modest to moderate associations with task‐based measures of cognitive functioning and impulsivity,^6^, ^61^, ^77^ many PD patients are indeed aware of alterations in cognitive functioning and behavioral changes.^78^

The use of first‐order factors provided specific information on domains most affected in patients with ICB, which may be of use when clinically evaluating a patient with PD for an ICB, and when considering future therapeutic interventions. It is useful to note that the BIS‐11 captures broad behavioral constructs, which is different than other assessments such as the QUIP, which is more limited to explicit behaviors that are commonly encountered in the clinical setting (e.g., eating, sexual activity, gambling, etc.). Here we show that patients with ICBs were significantly more impulsive, particularly in the attentional and non‐planning domains of self‐control and cognitive complexity. Due to the cross‐sectional nature of this study, it remains unclear if the increases in impulsivity are due to alterations from PD pathophysiology, secondary effects of chronic dopaminergic treatments, or both. Future studies investigating the relationship between ICBs in PD in a DAA naïve group may help to elucidate the role of DAA in development of ICBs. This study also reinforces the relevance of non‐motor symptoms in PD, as these findings emphasize the cognitive changes that may prove valuable in assessing the efficacy of a therapeutic intervention.

## Conflict of Interest

The authors do not report any conflicts of interest.

## Supporting information


**Appendix S1** **.** Description of BIS‐11 factor structure.
**Table S1** **.** Demonstration of the first‐ and second‐order factor structure, as well as item scoring distribution for each subscale of the BIS‐11.
**Table S2** **.** General Linear Model controlling for age, sex, LEDD and MoCA scores.
**Data S1** **.** R code used for general linear model statistical analyses
