# Supplementary material for: Self‐reported rates of impulsivity in Parkinson’s Disease
Source: Ann Clin Transl Neurol. 2020 Mar 29;7(4):437–48. doi: 10.1002/acn3.51016 (PMC7187703; doi:10.1002/acn3.51016)
Supplement: Supplementary file 1 — Appendix S1 . Description of BIS‐11 factor structure. Table S1 . Demonstration of the first‐ and second‐order factor structure, as well as item scoring distribution for each subscale of the BIS‐11. Table S2 . General Linear Model controlling for age, sex, LEDD and MoCA scores. Data S1 . R code used for general linear model statistical analyses [file ACN3-7-437-s001.docx]

**Supplementary Information**

The current BIS-11 is a 30-item questionnaire that was derived from psychometric analyses of the previous 34-item BIS-10 questionnaire completed by a variety of age and disease populations including: undergraduate students, substance abuse patients, general psychiatry patients, and male prison inmates. A principal components analysis suggested six correlated first-order components (see Fig. 1 in paper for hierarchical structure). These subdomains include: 1) attention - the ability to focus well on a current task; 2) cognitive instability - having intruding thoughts that make tasks difficult to complete; 3) motor impulsiveness – acting quickly without consideration of potential consequences; 4) perseverance – keeping a stable lifestyle (such as job continuity or living in the same place); 5) cognitive complexity – enjoyment of mental challenges such as puzzles that require higher-order processing; 6) self-control – performs actions after careful consideration and with deliberateness. The questions from the BIS-11 that contribute to each of these sub-domains are shown in Supplementary Table 1.

**Table and Figure Legends**

**Supplementary Table 1.** Demonstration of the first- and second-order factor structure, as well as item scoring distribution for each subscale of the BIS-11.

**Supplementary Table 2.** General Linear Model controlling for age, sex, LEDD and MoCA scores. Results show significant differences in first-order self-control and cognitive complexity between ICB- and ICB+ subjects. The first-order attention domain is not significant between groups using this model.

**Supplementary Table 1.**

| **2nd Order Factors** | **1st Order Factors** | **# of** **items** | **Items contributing to each subscale** |
| --- | --- | --- | --- |
| Attentional | Attention | 5 | 5, 9*, 11, 20*, 28 |
|  | Cognitive Instability | 3 | 6, 24, 26 |
| Motor | Motor | 7 | 2, 3, 4, 17, 19, 22, 25 |
|  | Perseverance | 4 | 16, 21, 23, 30* |
| Nonplanning | Self-Control | 6 | 1*, 7*, 8*, 12*, 13*, 14 |
|  | Cognitive Complexity | 5 | 10*, 15*, 18, 27, 29* |
|  |  |  | *reverse scored items |

**Supplementary Table 2.**

| **BIS-11 Scores** | **P-value** | **P-corrected value** | **Significant** |
| --- | --- | --- | --- |
| Total | 0.0006 | 0.0019 | TRUE |
| First-Order Factors |  |  |  |
| Attention | 0.8939 | 0.8939 | FALSE |
| Cognitive Instability | 0.6758 | 0.751 | FALSE |
| Motor | 0.1239 | 0.2066 | FALSE |
| Perseverance | 0.2627 | 0.3283 | FALSE |
| Self-Control | 0.00003 | 0.0003 | TRUE |
| Cognitive Complexity | 0.0171 | 0.0343 | TRUE |

**R Code:**

form <- as.formula(paste(score[i],"~ICD + Age + sex + MoCA + LEDD"))

mod <- lm(form,data=data)
